# On-chip functional neuroimaging with mechanical stimulation in *Caenorhabditis elegans* larvae for studying development and neural circuits†
†Electronic supplementary information (ESI) available. See DOI: 10.1039/c7lc01201b


**DOI:** 10.1039/c7lc01201b

**Published:** 2018-01-09

**Authors:** Yongmin Cho, David N. Oakland, Sol Ah Lee, William R. Schafer, Hang Lu

**Affiliations:** a School of Chemical & Biomolecular Engineering , Georgia Institute of Technology , USA . Email: hang.lu@gatech.edu; b Medical Research Council Laboratory of Molecular Biology , Cambridge , UK

## Abstract

Mechanosensation is fundamentally important for the abilities of an organism to experience touch, hear sounds, and maintain balance. *Caenorhabditis elegans* is a powerful system for studying mechanosensation as this worm is well suited for *in vivo* functional imaging of neurons. Many years of research using labor-intensive methods have generated a wealth of knowledge about mechanosensation in *C. elegans*, and the recent microfluidic-based platforms continue to push the boundary for this field. However, developmental aspects of sensory biology, including mechanosensation, are still not fully understood. One current bottleneck is the difficulty in assaying larvae because they are much smaller than adult worms. Microfluidic devices with features small enough for larvae, especially actuators for the delivery of mechanical stimulation, are difficult to design and fabricate. Here, we present a series of automatic microfluidic platforms that allow for *in vivo* functional imaging of *C. elegans* responding to controlled mechanical stimulation at different developmental stages. Using a novel fabrication method, we designed highly deformable pneumatically actuated on-chip structures that can deliver mechanical stimulation to larval worms. The PDMS actuator allows for quantitatively controlled mechanical stimulation of both gentle and harsh touch neurons, by simply changing the actuation pressure, which makes this device easily translatable to other labs. We validated the design and utility of our systems with studies of the functional role of mechanosensory neurons in developing worms; we showed that gentle and harsh touch neurons function similarly in early larvae as they do in the adult stage, which would not have been possible previously. Finally, we investigated the effect of a sleep-like state on neuronal responses by imaging *C. elegans* in the lethargus state.

## Abstract 2

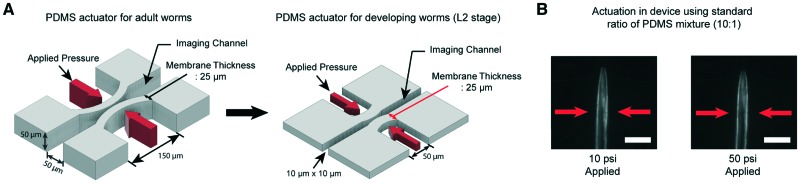
New designs of microfluidic devices can facilitate recording of *C. elegans* larvae neuronal responses to precise mechanical stimuli, which reveal new understanding of development of mechanosensory neurons and circuits.

## Introduction

Mechanosensation is one of the most important sensory modalities in an organism for interacting with the surrounding environment. Diverse mechanoreceptors transduce pressure to give rise to tactile and auditory sensations.^1^–^4^ The structure and functional organization of the nervous system for perception significantly changes during development. It is a critical time for the development of cognition, learning, and memory.^5^–^7^ Despite advances in the field of neuroimaging, surprisingly little is known regarding the neural basis of mechanosensation in developing nervous systems.

*Caenorhabditis elegans*, a free-living nematode, is an excellent model organism to study mechanosensation during development. Many of the genes known to govern both mechanotransduction and development are conserved from worms to humans.^8^–^19^ Furthermore, *C. elegans* has a relatively short developmental period (3 days) and a stereotyped and well-categorized cell lineage. The developmental origins of all 302 neurons in an adult hermaphrodite have been traced from zygote to adult. A combination of easy genetic manipulation and the optical transparency of the worm allows for non-intrusive *in vivo* imaging of neuronal activity.

In addition, *C. elegans* exhibits sleep-like states (lethargus) at the end of each larval stage.^20^,^21^ The expected duration and placement of these states in the worm's life are well documented and generally last around 1–2 hours at the completion of and transition to a new larval stage.^20^ Lethargus displays fundamental similarities to mammalian sleep, such as suppressed locomotion and feeding, a specific posture, and decreased responsiveness.^20^–^28^ Moreover, changes to synaptic connectivity are known to occur during lethargus periods,^29^–^31^ suggesting that sleep is necessary for healthy nervous system development. Therefore, *C. elegans* may also be a good model system to study the relationship between sleep-like states and developmental changes in the nervous system.

Previous systems have demonstrated a classic approach to the delivery of mechanical stimuli to adult worms and the optical or electrophysiological recording of neuronal responses using a piezo-driven micro-stylus.^16^,^32^–^36^ However, these systems require animals to be immobilized with glue, limiting the experimental throughput and preventing the recovery of animals for further study.

Recently, microfluidics has been applied to not only mimic conventional assays, but also deliver mechanical stimuli with greater precision and throughput of experiments on adult worms, compared to traditional methods.^37^–^40^ For example, our system demonstrated several advantages compared to traditional methods and other microfluidic platforms for *in vivo* functional imaging.^38^ First, the platform could stimulate both gentle and harsh touch neurons with single pulse mechanical stimulation. Second, it could elicit a tuneable response from both gentle and harsh touch receptor neurons depending on the strength of the stimulus delivered. Third, our device could be operated automatically, allowing for a high-throughput drug screen for small molecules that affect mechanosensation. However, these microfluidic-based systems (including ours) only permitted the recording of neuronal activity in adult worms. This is because delivering controlled mechanical stimuli to and handling smaller worms are difficult. With elegant agarose hydrogel microcompartments,^28^,^41^ neuronal responses of ALM or AVA to tapping stimuli during early developmental stages or in sleep-like states have been observed; however, responses in these and other neurons to controlled stimuli (strength and duration), with precision of the stimulation position, are unknown.

Since the size of larval worms is smaller than that of adult ones, all of the structures in the microfluidic systems, including the actuator, should be reduced in size compared to the device for adult worms. However, the development of a highly deformable PDMS membrane structure on a smaller scale proved to be difficult. Specifically, while the width and height of the actuated PDMS membrane should be decreased to accommodate smaller larvae, the same membrane thickness as the device for adult worms (25 μm) had to be used to ensure a highly successful bonding rate and create an actuator working robustly in an realistic operating pressure range. As a result, the reduced aspect ratio of the width and height to the thickness of the actuators would require much higher pressure to achieve deformation similar to that of the adult platform. These pressures would have operated outside a practical pressure range.

Here, we present a series of microfluidic platforms that can record neuronal responses to mechanical stimuli in early larval stages. By using a hybrid device fabrication method, we have created easily deformable PDMS structure that can deliver controlled mechanical stimuli to developing worms. As before,^38^ we demonstrated that these platforms can greatly improve the throughput and robustness of experiments compared to conventional *in vivo* functional imaging methods by coupling them with an automation step. We validated the design and utility of these systems by recording neuronal activities in developing *C. elegans*. We show that worms in the L2 stage used the same functional mechanosensory circuits as those in the adult stage. Moreover, we show that in comparing the response to mechanical stimuli during sleep and awake periods, worms have highly reduced neuronal activity in the lethargus state.

## Materials and methods

### Strains

*C. elegans* were maintained under standard conditions and fed OP50 bacteria.^42^ The following strains were used in this study:

AQ3236 *ljSi2[mec-7::GCaMP6m::SL2TagRFP, unc-119] II; unc-119(ed3)*, TV21149 *wyls587[ser-2prom3::mCherry::unc-54 3′UTR, ord-1::GFP]; wyls5007[ser-2prom3::GCaMP6, elg-17::mCherry]*, ZM9059 *hpIs580[rig-3::GCAMP6::mCherry], RW1596 stEx30[myo-3::GFP + rol-6(su1006)]*, GT247 *mec-4(e1611); ljSi2[mec-7::GCaMP6m::SL2TagRFP, unc-119] II; unc-119(ed3)*, GT253 *mec-4(u253); ljSi2[mec-7::GCaMP6m::SL2TagRFP, unc-119] II; unc-119(ed3),* CX11935 *kyEx3252[Pstr-2:GCaMP3.0 Pofm-1:GFP]*.

A hatch-off procedure was used to synchronize larvae.^43^ Briefly, adult hermaphrodites laid eggs overnight at 20 °C on NGM plates. Hatched larvae and adults were removed from the plates with three successive washes with M9 buffer. The embryos remaining on the plates were incubated at 20 °C for only 1 hour. Highly synchronized L1 larvae that hatched in the 1 hour window were washed off and transferred to new NGM plates.

### Chip design and fabrication

The devices consist of a worm inlet/outlet, an imaging channel (10–25 μm wide and high, depending on the developmental stage),^44^ and four sets of actuated PDMS membranes. Animals are loaded into the imaging channel using a set of actuated PDMS membranes. Then, they are trapped, but not physically restricted, in the imaging area by another set of actuated membranes. The width of the actuated PDMS membrane that comes into contact with the worm is 50 μm. The distance between the first and second sets of membranes is 50–75 μm and that between the second and third sets of membranes is 75–200 μm, depending on the developmental stage.

To create the easily actuated PDMS structure to stimulate and trap worms, a 30 : 1 ratio of the PDMS monomer and a curing agent (Sylgard 184, Dow Corning) was deposited *via* spin coating to create a thin layer for the bottom feature layer (speed: 1000 rpm, ramp: 5 s, and spin time: 30 s). Separately, a 10 : 1 PDMS mixture was directly poured onto a blank master, which does not have any features, to create a thick and mechanically rigid handle layer. Both PDMS layers were then placed into an 80 °C oven for 25–30 minutes until they were rigid but sticky. After they were manually aligned, an additional 10 : 1 PDMS mixture was poured on top of the layers to fill the gap between the aligned layers and the Petri dish, and then the Petri dish was placed into an 80 °C oven overnight.

After curing, PDMS devices were cut to size, and featured PDMS chunks were created *via* puncturing the cured PDMS with sharpened gauge needles (18 gauge for worm in- and outlet and 19 gauge for all valves). The prepared devices were then cleaned with scotch tape and exposed to air plasma for 15 seconds before being placed and covalently bonded to a glass surface to create closed channels. Right after this bonding procedure, the devices were placed on top of a 150 °C hot plate for 3 min to increase the adhesion between PDMS and the cover glass.

### Simulation of mechanical performance of the PDMS membrane

The deformation of the PDMS membrane was simulated using COMSOL Multiphysics, a commercial software package for modeling and simulating physics-based problems using the finite element method (FEM). The geometry of the PDMS membrane structure for stimulation followed the fabricated L2 device parameters (50 μm width, 13.5 μm height, and 25 μm thickness). To simulate the deformation of the 10 : 1 standard PDMS mixture, a Young's modulus of 1100 kPa was used. For the simulation of the 30 : 1 PDMS mixture, a Young's modulus of 110 kPa was used.^45^

### Calcium imaging

All imaging experiments were performed on a Leica DMIRB inverted microscope using a 40× air objective (N.A. = 0.75). Video sequences were captured using a Hamamatsu EM-CCD camera with 100 ms exposure time. Simultaneous dual color imaging was performed using a DV2 beam splitter (Photometrics) containing a GFP/RFP filter set. Excitation light for fluorescence imaging was delivered through a projector system.^46^ In experiments for the measurement of mechanosensory neuronal responses, stimuli were delivered 5–10 s after recording the baseline activity of the neurons. Videos were recorded for 10–90 s following stimulus delivery.

### Determining the lethargus state for calcium imaging

We identified animals in the lethargus state by monitoring worm behavior under a dissecting scope. As previously reported, lethargic animals exhibit either complete quiescence or a specific pattern of quiescence interspersed with short bouts of active movement.^20^,^25^,^26^ When a worm met these criteria, it was classified as lethargic and transferred to a worm loading reservoir. After loading individual worms into the imaging channel of the device, we waited for 5 min to ensure that the worm remained in the lethargus state before recording the neuronal activity.

### Data analysis

Fluorescence intensities for each frame were extracted using a customized neuron-tracking MATLAB script.^38^ In strains where both GCaMP6 and RFP were expressed, the ratio between intensity values was computed (*R* = *I*_Green_ROI_/*I*_Red_ROI_) in order to minimize movement artifacts. When only GCaMP was available, fluorescence values were computed by subtracting the background intensity (*F* = *I*_Green_ROI_ – *I*_Green_Back_). GCaMP and RFP intensities were measured as the mean pixel intensity of the 25 brightest pixels in a circular region of interest (ROI) with a 5 pixel radius. Calcium traces were computed as the change in *R* or *F* from the baseline value (Δ*R*/*R*_o_ = (*R* – *R*_o_)/*R*_o_ or Δ*R*/*R*_o_ = (*F* – *F*_o_)/*F*_o_). Baseline values were computed as the mean *R* prior to stimulus delivery.

## Results and discussion

**Fig. 1:**
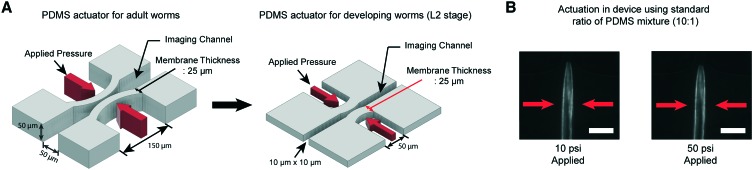
A key engineering challenge is the scaling-down of the device for adult worms^38^ to match the size of the imaging channel to the size of developing worms. (A) Schematic diagrams of the PDMS actuator for the delivery of mechanical stimulation for adult (left) and L2 stage worms (right). All scales of the device for adult worms have to be scaled down except the thickness of the PDMS membrane (25 μm) for L2 stage imaging to minimize the worm movement and deliver localized mechanical stimulation. (B) Example images of worms in the device made by using a standard ratio of the PDMS mixture (10 : 1). 10 psi (left) and 50 psi (right) are applied by using anterior touch valves (red arrows). Worms were cultured 20 hours after hatching. Scale bar: 25 μm.

We have developed microfluidic devices that deliver precise and robust mechanical stimuli to developing worms. While the overall design is similar to our previous device,^38^ the dimensions of the structures have been reduced to accommodate much smaller larval worms (Fig. 1A). If a worm's body is well fitted to the size of the imaging channel, its movement can be highly reduced, which allows high-quality imaging. For adult worms, the channel width is about 50–60 μm, depending on age, while for L2, it is 10 μm wide.

Moreover, *C. elegans* has two different receptive fields of mechanosensory neurons: the anterior and posterior regions of the body.^47^ As before,^38^ our platform was designed to deliver precise, localized “touches” in order to stimulate distinct neurons. For the delivery of localized stimulation to small larvae (the length of L2 animals is ∼360–380 μm), the width of the actuated PDMS membrane that comes into contact with the worm is reduced to 50 μm from 150 μm for adult worms (the length of adults is ∼1110–1150 μm).^48^ Using the larger 150 μm width actuator for L2 worms would result in both the anterior and posterior touch circuits being activated.

Even though all the dimensions of the structure have been reduced, the thickness of the PDMS membrane (25 μm) had to be kept the same as that in the device for adult worms to ensure a high success rate in bonding and create an actuator working in a realistic operating pressure range. If we decrease the membrane thickness, the area available for bonding to the cover glass is reduced, resulting in the devices rupturing when we apply pressure to actuate the structures. Retaining the same membrane thickness, however, dramatically reduces the aspect ratio of the width and height to the thickness of the actuators (Fig. 1A). This would lead to an increase in the pressure needed to deform the membranes, quickly reaching an impractical point. As shown in Fig. 1B, even at 50 psi, the membrane deformation is negligible and not sufficient to activate any of the mechanoreceptor neurons in *C. elegans* larvae (data not shown).

**Fig. 2:**
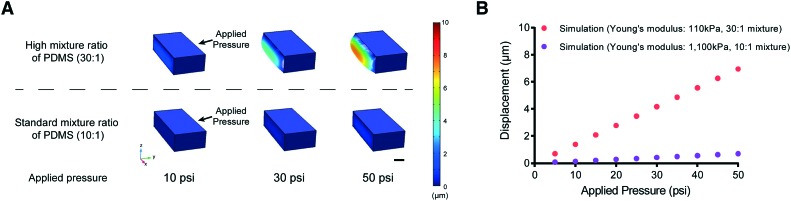
Simulation results for the displacement of the actuated membrane by applying various pressures. (A) Examples of PDMS membrane deformation using different applied pressures from the results of COMSOL simulation. The color bar represents the scale of membrane displacement. Scale bar: 10 μm. (B) Red and purple represent simulation results from COMSOL Multiphysics (Young's modulus – red (30 : 1 PDMS mixture used in this study: 110 kPa) and purple (10 : 1 standard PDMS mixture: 1100 kPa)).

One possible approach to overcome this problem of PDMS membrane deformability is to decrease its elastic modulus (by reducing the amount of cross-linker in the PDMS mixture, for example). We tested this idea using computational modeling (COMSOL Multiphysics, see Materials and methods) to simulate the deformability of the membrane designed to fit the size of L2 stage worms at various applied pressures (Fig. 2). The simulation suggested that a higher ratio of PDMS to cross-linker (30 : 1) leads to sufficient deformability of the PDMS structure for L2 stage worms. In comparison, the membrane made with the standard ratio of PDMS to cross-linker (10 : 1) is not deformed even at high pressures (>50 psi) (Fig. 2B).

**Fig. 3:**
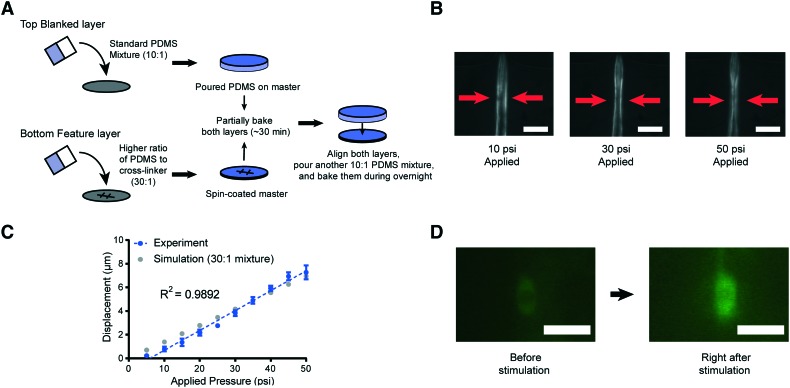
Schematic of the new device fabrication method and results of membrane displacement measurements. (A) Schematic of the new fabrication method. (B) Example images of worms in the device made by using a high ratio of the PDMS mixture (30 : 1). 10 psi (left), 30 psi (middle), and 50 psi (right) are applied by using anterior touch valves (red arrows). Worms were cultured 20 hours after hatching. Scale bar: 25 μm. (C) Displacement of the actuated membrane by applying pressures. Experimental (blue) and simulation results (gray, the same as red in Fig. 2B). The *R*-square value is 0.9892. (D) Sample frames from an activated ALM neuron show changes in fluorescence due to mechanical stimuli (scale bar: 5 μm, used 100× magnification).

Decreasing the elastic modulus of the PDMS results in additional design challenges for our platforms. One potential source of challenges is that the use of a higher ratio of the PDMS mixture for the entire device would affect its handling and operability. To address this issue, we developed a hybrid fabrication method that simultaneously creates a more deformable membrane structure while maintaining the necessary mechanical rigidity of the overall device structure (Fig. 3A). Specifically, the high-ratio PDMS mixture (30 : 1) is spin coated onto the featured master wafer for a uniform and consistent thickness of the layer. Generating a uniform thickness is critical for reproducing devices with the same pressure-to-deformation ratio. Separately, the standard 10 : 1 PDMS polymer mixture is poured onto a blank wafer in order to make the entire device thick and mechanically rigid for the connection with tubing *via* metal pins. Both layers are partially cured for ∼30 minutes at 80 °C, which renders the PDMS solid enough for alignment of the two layers. Using two solid layers in this process is important, as the cross-linker would permeate from the top to the bottom layer in the liquid form, which would reduce the ratio of PDMS to cross-linker and increase the rigidity of the PDMS membrane.

To examine the reproducibility and robustness of the membrane deformation, we measured its displacement using different applied pressures and took images from transgenic worms that express GFP along the body-wall muscle in our device for L2 stage worms (Fig. 3B and C). We observe no obvious deformation of the worms' body wall when using a low applied pressure of 10 psi to the anterior touch valve (left panel of Fig. 3B). However, as we increase the pressure, we observe a corresponding increase in the deformation of the body wall in that specific region (middle and right panels of Fig. 3B). Experimental measurements over a large range of pressures show that the deformation in the body wall is linear to the actuation pressure (Fig. 3C). Remarkably, this trend agrees with the predictions of our computational modeling, where the only changeable variable was the elastic modulus of the PDMS (Fig. 3C). This linear relationship makes the strength of mechanical stimulation easy to control *via* applied pressure. Moreover, the error in the measurement of membrane displacement is small, which indicates that the mechanical stimulation delivered by our platforms is highly reproducible. In addition to the direct measurement of the tissue deformation, we also measured the activation of mechanoreceptor neurons labeled with a calcium indicator. Upon well-controlled deformation of the PDMS membrane, the gentle touch neuron ALM is activated in an L2 worm (Fig. 3D). Not surprisingly, the chemosensory neuron, AWC, does not show response to typical mechanical stimuli in our system (Fig. S1†).

To enhance the viability and robustness of mechanostimulation on *C. elegans* larvae, we proceeded to automate the operation of the platform as with the device for adult worms.^38^ This greatly improves the experimental throughput, while minimizing the variability introduced by human handling/manipulation. Specifically, all actuated valves were connected to a pressure source *via* individually controlled off-chip solenoid valves. This allows for automated and rapid operation with a custom MATLAB script:^38^ the automated worm loading/unloading procedure takes only a few seconds and experiment duration ranges from several seconds to a few minutes depending on the assay. Therefore, experimental throughput is only limited by the length of the assay. Since our synchronization protocol typically yields several hundred worms (see Materials and methods), up to ∼100 worms per hour can be assayed in this new platform.

Another important advantage of our device is that worms' neurons can be easily imaged and tracked, as the animals have limited movement in the channel. Both the width and height of the imaging channel and trapping valves minimize the movement of worms, allowing high-quality calcium imaging without using other immobilization techniques that may affect neuronal responses. Because we want to image worms at different developmental stages of their lives, we designed devices ranging from 10 μm to 25 μm in width. A series of devices were optimized for worms at each developmental stage^20^ to minimize the worm's movement within the channel. Table 1 summarises the ideal post-hatching developmental time in hours for each of our device sizes. Thus, by using a set of our devices, we can monitor the activity of neurons in mechanosensory circuits from early larvae to adult worms.

**Table 1:** The channel sizes of devices were optimized for minimal movement within the channel. This table shows the ideal developmental time, in hours, for each of our device sizes

Time after hatching (h)	18–24 h (20 °C)	28–34 h (20 °C)	38–42 h (20 °C)	46–50 h (20 °C)
Channel width	10 μm	15 μm	20 μm	25 μm

To demonstrate the utility of the system, we conducted *in vivo* functional imaging experiments. First, we asked how the functional role of touch receptor neurons changed during development. Studies reported previously using conventional methods, due to technical challenges, have not directly demonstrated neuronal activation in larvae in response to mechanical stimuli. In a previous study, we observed that both gentle and harsh touch receptor neurons in adult worms clearly distinguished the difference in magnitudes of an applied mechanical stimulation.^38^ We asked whether these neurons are functionally developed as early as the L2 stage, and whether they exhibit graded responses as in the adult stage.

**Fig. 4:**
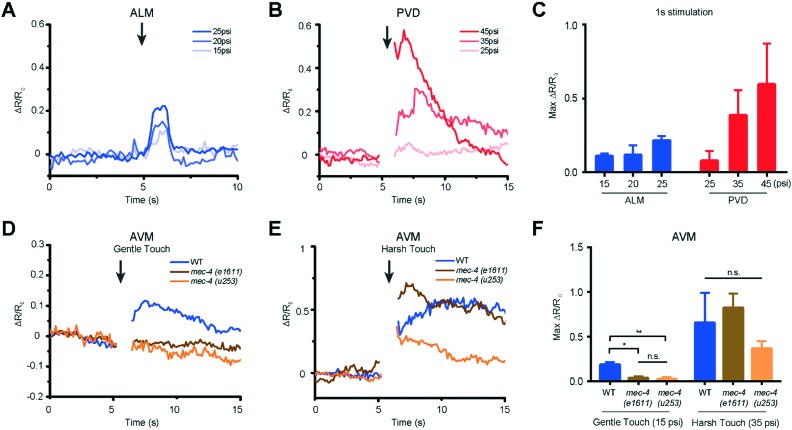
Both gentle and harsh touch receptor neurons can distinguish the magnitude of applied mechanical stimulation. (A) ALM responses to anterior touch with 1 s and diverse pressures (15 psi: *n* = 9, 20 psi: *n* = 7, 25 psi: *n* = 10). (B) PVD responses to posterior touch with 1 s and diverse pressures (25 psi: *n* = 5, 35 psi: *n* = 13, 45 psi: *n* = 5). (C) Maximum calcium responses of ALM and PVD to a variety of applied pressures. (D) AVM responses of wild-type and *mec-4* (*e1611* and *u253*) mutants to anterior gentle touch (1 s and 15 psi, WT: *n* = 11, *mec-4* (*e1611*): *n* = 7, *mec-4* (*u253*): *n* = 15). (E) AVM responses of wild-type and *mec-4* (*e1611* and *u253*) mutants to anterior harsh touch (1 s and 35 psi, WT: *n* = 7, *mec-4* (*e1611*): *n* = 5, *mec-4* (*u253*): *n* = 13). (F) Maximum calcium responses of wild-type and *mec-4* mutant animals (Kruskal–Wallis test, *p < 0.05, **p < 0.01, n.s. is non-significant). (A–F) All worms in these experiments were cultured 18–22 h after hatching.

We used our system to measure the responses of both gentle (AVM, ALML/R, PVM, and PLML/R) and harsh touch (PVD) receptor neurons at different extents of deformation or different stimulation durations. The critical parameter in mechanosensory stimulation is displacement, not force.^33^ Our design has the advantage of simplicity in operation to deliver both gentle and harsh touches, because the deflection of the PDMS actuator is directly proportional to the applied pressure. We show for the first time that ALM neurons of L2 animals (18–22 hours after hatching) can respond to anterior stimuli at varying levels of pressure and duration (Fig. 4A and S2 and Movie S1†). The strong mechanical stimulus (25 psi) can elicit higher calcium transient peaks in ALM neurons compared to the weak stimulus (15 psi). (Fig. 4A and C and S2†).

Similarly, we delivered posterior stimuli of varying pressure and duration and observed the response in PVD neurons in L2 larvae (Fig. 4B and S2 and Movie S2†). As in the adult animals,^38^ PVD required stronger mechanical stimulation to be activated than gentle touch neurons. PVD had a negligible response to stimuli in the gentle touch regime (lower than 25 psi, data not shown). On the other hand, in the harsh touch regime, either a longer duration of stimuli (1 s) at low pressure (35 psi) or a shorter duration of stimuli at high pressure (45 psi) can elicit PVD neuronal response (Fig. 4B and C and S2†). Interestingly, it is known that PVD does not grow its multidendritic processes until L3 and L4; in the L2 stage, PVD has only a simple longitudinal primary dendrite.^49^ Our data suggest that these anatomical specializations are not required for harsh touch sensation. Beyond L2, both gentle and harsh touch neurons continue to respond to controlled mechanical stimuli (Fig. S3 and S4†).

Another gentle touch neuron of interest is AVM. Previously, based on behavioural assay in combination with laser microsurgery, AVM's function was detectable at 35–40 hours after hatching (*e.g.* late L3) and was assumed to be not functionally developed in L2.^50^ We tested AVM activities directly *via* functional imaging and found that AVM can indeed respond to mechanical stimulation in L2, in both gentle and harsh touch regimes (Fig. 4D and E and Movie S3†). This suggests that AVM itself is sensitive to mechanical stimulation in L2, but might not be functionally wired into the escape circuit yet. We also measured the response of the AVM neuron in mutants with specific defects in gentle touch. The *mec-4* gene encodes a DEG/ENaC channel; *u253* is a null allele that only affects gentle touch sensation while *e1611* is a dominant allele that causes necrotic neurodegeneration in older worms.^32^,^50^ Although insensitive to stimulation in the gentle touch regime (anterior touch with 1 s and 15 psi), the *mec-4* mutants were responsive to stimulation in the harsh touch regime (anterior touch with 1 s and 35 psi) (Fig. 4D–F), consistent with previous reports in adult worms.^32^,^38^ The observation that the *mec-4* mutants show defects may indicate that the MEC-4 protein is essential for touch neuron activity in response to gentle touch in the L2 stage of the worm. Thus, the data suggest that the functional role of mechanosensory neurons is already developed by the L2 stage and similar to that in the adult stage.

**Fig. 5:**
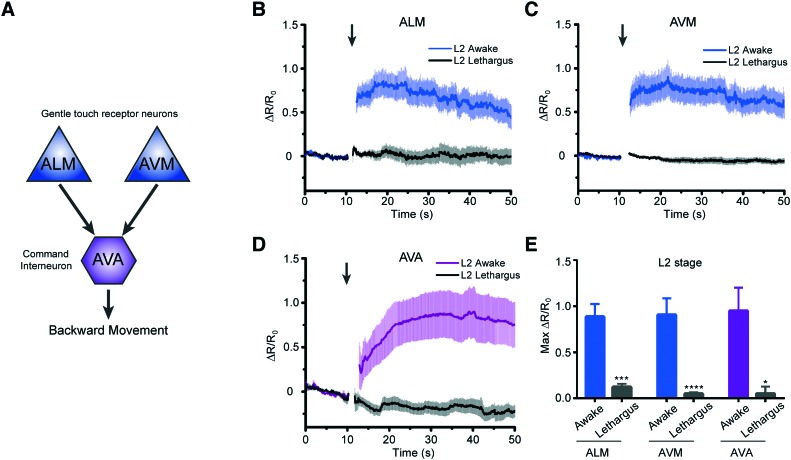
Worms in the L2 lethargus state show drastically reduced neural responsiveness to mechanical stimulation. (A) Simplified circuit diagram showing two mechanosensory neurons connecting to AVA command interneuron to backward locomotion behavior. (B–D) Average traces of calcium responses of L2 and L2 lethargus worms in (B) ALM (L2: *n* = 19, L2 lethargus: *n* = 9), (C) AVM (L2: *n* = 19, L2 lethargus: *n* = 15), and (D) AVA (L2: *n* = 15, L2 lethargus: *n* = 13) to 1 s anterior touch with 50 psi. (E) Maximum calcium responses of L2 and L2 lethargus worms (Kruskal–Wallis test, *p < 0.05, **p < 0.01, ***p < 0.001, ****p < 0.001). (B–E) All worms in these experiments were cultured either for 18–22 h for L2 or 24–25 h for L2 lethargus. Error bars represent SEM.

We next asked how the neuronal response to mechanical stimuli is modulated based on physiological states in developing worms. *C. elegans* normally passes through four larval stages, L1 to L4, to reach adulthood. At the end of each developmental stage, the worm enters the lethargus state.^20^,^21^,^24^ However, due to the limitation of techniques, most of the *in vivo* imaging in lethargus has thus far been done with larger L4 stage animals.^23^,^24^,^27^ Although Schwarz *et al.* observed reduced ALM responses to tapping stimuli in the sleep-like state,^28^ it is still unknown whether other neurons such as touch neuron AVM and interneuron AVA in mechanosensory circuits remain responsive during lethargus in early larvae. To investigate the behavior of these circuits, we measured the response of two sensory neurons, ALM and AVM, and the interneuron AVA to mechanical stimuli in awake L2 animals (18–22 hours after hatching) *versus* lethargic L2 animals (24–25 hours after hatching) (Fig. 5A). In order to reduce stochastic effects on the neuronal response, we applied a strong mechanical stimulus (50 psi) to the anterior region and recorded the calcium transients (Fig. 5B–E). As seen in Fig. 5, even at this extremely strong stimulation, lethargic worms elicit almost no response. In contrast, the activities of neurons are clear in the awake worms and show a large response. Interestingly, the effect of L4 lethargus (about 50% reduction in touch responses compared to L4 or adult)^27^ was much less dramatic than the effect of L2 lethargus on touch-evoked ALM responses. The reduced neuronal responses are seen in both gentle touch neurons, ALM and AVM, along with the interneuron, AVA (Fig. 5B–E). While this phenomenon has been observed before at the behavioral level,^20^ our data further demonstrate that mechanosensory neurons display reduced sensitivity to stimuli in the lethargus state. Therefore, this demonstrates that our device can be used to study the sleep states of *C. elegans*, which is difficult to do using conventional methods.

Together, these experiments demonstrate that we can activate distinct types of mechanosensory neurons by simply changing the magnitude of applied pressure in our system. This technical development allowed us to make novel observations into early development and functional analysis of the mechanosensory neurons.

## Conclusions

*In vivo* functional imaging of *C. elegans* in early larval stages is currently limited, because conventional methods do not have the level of precision that is needed. We have developed a range of microfluidic platforms that allow for precise interrogation of mechanosensation in *C. elegans* at several life stages, ranging from the early larval stage to late adulthood. These devices combine the ability to robustly perform high-quality functional imaging with the ability to deliver a variety of mechanical stimuli to different regions of the worm's body. Using these platforms, we observed how the functional role of both gentle and harsh touch neurons in mechanosensory circuits changes from developing to adult worms. Specifically, AVM, which was known to be functionally developed in the L3 stage based on behavioral studies, shows neuronal responses to mechanical stimulation even in the L2 stage. Both gentle and harsh touch neurons show graded responses to various mechanical stimuli. Moreover, we observed that the activity of neurons in mechanosensory circuits is reduced in the L2 lethargus state, compared to the awake L2 stage.

With access to highly improved throughput and more standardisable assays in our platforms, compared to conventional methods, it is now possible to develop high-throughput assays that screen genes or drugs for their effects on neuronal activity of mechanosensory circuits during the developmental period of *C. elegans*. We envision that this system will allow the study of both the fundamental biology of mechanosensation and the role of development in the formation of mechanosensory circuits. Finally, we expect that our platform greatly accelerates the discovery of drugs that target diseases linked to impaired mechanosensation, such as deafness.

## Conflicts of interest

There are no conflicts to declare.

## Supplementary Material

Supplementary information

Supplementary movie

Supplementary movie

Supplementary movie

## References

[cit1] Corey D. P. (2003). Neuron.

[cit2] Kung C. (2005). Nature.

[cit3] Kurima K., Yang Y., Sorber K., Griffith A. J. (2003). Genomics.

[cit4] Kawashima Y., Géléoc G. S., Kurima K., Labay V., Lelli A., Asai Y., Makishima T., Wu D. K., Della Santina C. C., Holt J. R. (2011). J. Clin. Invest..

[cit5] Giedd J. N., Blumenthal J., Jeffries N. O., Castellanos F. X., Liu H., Zijdenbos A., Paus T., Evans A. C., Rapoport J. L. (1999). Nat. Neurosci..

[cit6] Casey B., Tottenham N., Liston C., Durston S. (2005). Trends Cognit. Sci..

[cit7] Jin X., Pokala N., Bargmann C. I. (2016). Cell.

[cit8] Chalfie M., Sulston J. E., White J. G., Southgate E., Thomson J. N., Brenner S. (1985). J. Neurosci..

[cit9] Chalfie M., Au M. (1989). Science.

[cit10] Hong K., Driscoll M. (1994). Nature.

[cit11] Huang M., Chalfie M. (1994). Nature.

[cit12] Corey D. P., Garcia-Anoveros J. (1996). Science.

[cit13] Tavernarakis N., Shreffler W., Wang S., Driscoll M. (1997). Neuron.

[cit14] Tobin D. M., Madsen D. M., Kahn-Kirby A., Peckol E. L., Moulder G., Barstead R., Maricq A. V., Bargmann C. I. (2002). Neuron.

[cit15] Goodman M. B., Ernstrom G. G., Chelur D. S., O'Hagan R., Yao C. A., Chalfie M. (2002). Nature.

[cit16] O'Hagan R., Chalfie M., Goodman M. B. (2005). Nat. Neurosci..

[cit17] Kindt K. S., Viswanath V., Macpherson L., Quast K., Hu H., Patapoutian A., Schafer W. R. (2007). Nat. Neurosci..

[cit18] Chatzigeorgiou M., Yoo S., Watson J. D., Lee W.-H., Spencer W. C., Kindt K. S., Hwang S. W., Miller III D. M., Treinin M., Driscoll M. (2010). Nat. Neurosci..

[cit19] Delmas P., Coste B. (2013). Cell.

[cit20] Raizen D. M., Zimmerman J. E., Maycock M. H., Ta U. D., You Y.-J., Sundaram M. V., Pack A. I. (2008). Nature.

[cit21] Iwanir S., Tramm N., Nagy S., Wright C., Ish D., Biron D. (2013). Sleep.

[cit22] Van Buskirk C., Sternberg P. W. (2007). Nat. Neurosci..

[cit23] Cho J. Y., Sternberg P. W. (2014). Cell.

[cit24] Nichols A. L., Eichler T., Latham R., Zimmer M. (2017). Science.

[cit25] Nagy S., Tramm N., Sanders J., Iwanir S., Shirley I. A., Levine E., Biron D. (2014). Elife.

[cit26] Nagy S., Raizen D. M., Biron D. (2014). Methods.

[cit27] Choi S., Chatzigeorgiou M., Taylor K. P., Schafer W. R., Kaplan J. M. (2013). Neuron.

[cit28] Schwarz J., Lewandrowski I., Bringmann H. (2011). Curr. Biol..

[cit29] White J., Albertson D., Anness M. (1978). Nature.

[cit30] Hallam S. J., Jin Y. (1998). Nature.

[cit31] Tramm N., Oppenheimer N., Nagy S., Efrati E., Biron D. (2014). PLoS One.

[cit32] Suzuki H., Kerr R., Bianchi L., Frøkjaer-Jensen C., Slone D., Xue J., Gerstbrein B., Driscoll M., Schafer W. R. (2003). Neuron.

[cit33] Eastwood A. L., Sanzeni A., Petzold B. C., Park S.-J., Vergassola M., Pruitt B. L., Goodman M. B. (2015). Proc. Natl. Acad. Sci. U. S. A..

[cit34] Park S.-J., Goodman M. B., Pruitt B. L. (2007). Proc. Natl. Acad. Sci. U. S. A..

[cit35] Geffeney S. L., Cueva J. G., Glauser D. A., Doll J. C., Lee T. H.-C., Montoya M., Karania S., Garakani A. M., Pruitt B. L., Goodman M. B. (2011). Neuron.

[cit36] Petzold B. C., Park S.-J., Mazzochette E. A., Goodman M. B., Pruitt B. L. (2013). Integr. Biol..

[cit37] Cho Y., Hwang H., Porto D. and Lu H., presented in part at the 19th International Conference on Miniaturized Systems for Chemistry and Life Sciences (MicroTAS 2015), Gyeongju, Korea, October 25–29, 2015.

[cit38] Cho Y., Porto D., Hwang H., Grundy L., Schafer W. R., Lu H. (2017). Lab Chip.

[cit39] Nekimken A. L., Fehlauer H., Kim A. A., Manosalvas-Kjono S. N., Ladpli P., Memon F., Gopisetty D., Sanchez V., Goodman M. B., Pruitt B. L. (2017). Lab Chip.

[cit40] McClanahan P. D., Xu J. H., Fang-Yen C. (2017). Integr. Biol..

[cit41] Turek M., Besseling J., Bringmann H. (2015). J. Visualized Exp..

[cit42] Brenner S. (1974). Genetics.

[cit43] Hope I. A., C. elegans: a practical approach, OUP Oxford, 1999.

[cit44] Maguire S. M., Clark C. M., Nunnari J., Pirri J. K., Alkema M. J. (2011). Curr. Biol..

[cit45] Ochsner M., Dusseiller M. R., Grandin H. M., Luna-Morris S., Textor M., Vogel V., Smith M. L. (2007). Lab Chip.

[cit46] Stirman J. N., Crane M. M., Husson S. J., Wabnig S., Schultheis C., Gottschalk A., Lu H. (2011). Nat. Methods.

[cit47] Goodman M. B., WormBook: the online review of C. elegans biology, 2006, pp. 1–14. 10.1895/wormbook.1.62.1 PMC2806189 18050466

[cit48] Hall D. and Altun Z., C. elegans Atlas. Cold Spring Harbor Laboratory Press, Cold Spring Harbor, New York, 2008, pp. 1–15.

[cit49] Oren-Suissa M., Hall D. H., Treinin M., Shemer G., Podbilewicz B. (2010). Science.

[cit50] Chalfie M., Sulston J. (1981). Dev. Biol..

