# Supplementary material for: On-chip functional neuroimaging with mechanical stimulation in Caenorhabditis elegans larvae for studying development and neural circuits
Source: Lab Chip. 2018 Jan 9;18(4):601–9. doi: 10.1039/c7lc01201b (PMC5885276; doi:10.1039/c7lc01201b)
Supplement: Supplementary file 1 [file LC-018-C7LC01201B-s001.pdf]

Electronic Supplementary Material (ESI) for Lab on a Chip.
This journal is © The Royal Society of Chemistry 2018

Supplemental Figures

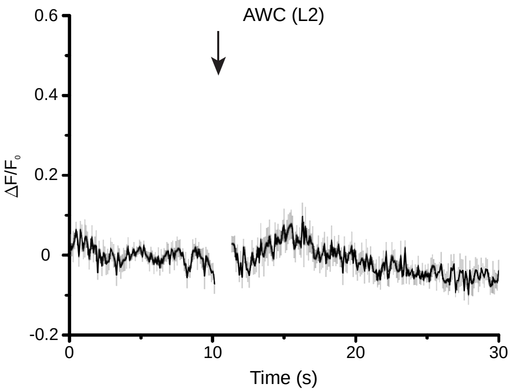

Fig. S1 Calcium dynamics of AWC neuron to 1s anterior stimulation at 25 psi (n=9). Arrow represents the time when mechanical stimulus delivered. Error bars represent SEM. All worms in these experiments were cultured 20-22h at 20 °C after hatch.

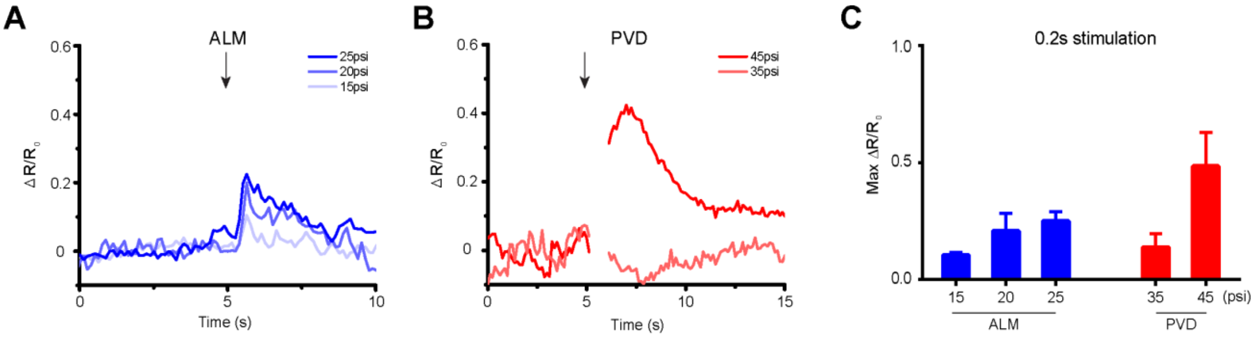

Fig. S2. Both gentle and harsh touch receptor neurons can distinguish the change of magnitude of applied mechanical stimulation.

(A) Average traces of calcium signals in ALM in response to anterior touch with 200ms and diverse pressure. (15 psi: n=6, 20 psi: n=6, 25psi: n=8). (B) Average traces of calcium signals in PVD in response to posterior touch with 200ms and diverse pressure. (35 psi: n=4, 45 psi: n=5). (C) Maximum responses of calcium transients in ALM and PVD neurons correlate with the applied pressure (0.2s stimulus, 15 to 45psi). (A-C) All worms in these experiments were cultured 18-22h at 20 °C after hatch.

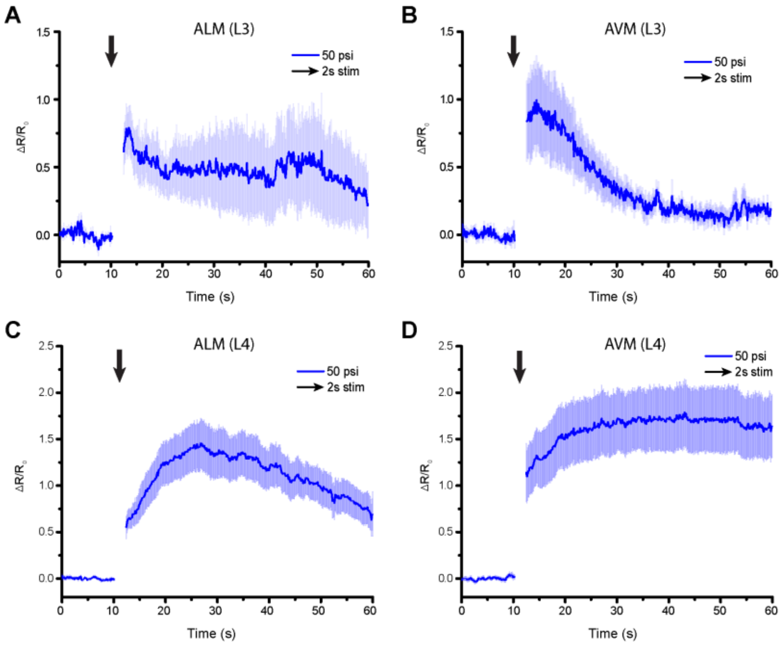

Fig. S3. Responses of gentle touch neurons to strong mechanical stimulations at different developmental stages.

(A-B) Average traces of calcium responses of L3 worms in gentle touch neurons. All worms in these experiments were cultured 30-32h at 20 °C after hatch. (A) ALM (n=8) and (B) AVM responses (n=7) to 2s anterior touch with 50 psi. (C-D) Average traces of calcium responses of L4 worms in ALM and AVM. All worms in these experiments were cultured 38-42h at 20 °C after hatch. (C) ALM (n=14) and (D) AVM (n=14) responses to 2s anterior touch with 50 psi. Error bars represent SEM.

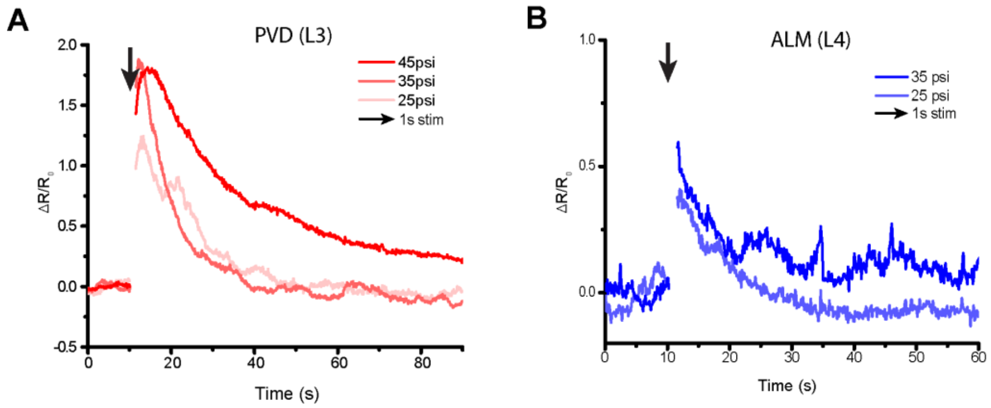

Fig. S4. Responses of both gentle and harsh touch neuron to various mechanical stimulations at different developmental stages.

(A) PVD responses in L3 stage to 1s posterior touch with various applied pressures (45 psi: n=14, 35 psi: n=6, 25 psi: n=5). (B) ALM responses in L4 stage to 1s anterior touch with various applied pressures (35 psi: n=4, 25 psi: n=5).

Movie 1: Calcium dynamics of ALM neuron to 1s anterior stimulation at 20 psi. Stimulus was delivered 10s after recording baseline of neuronal activity. The transgenic animal shown here expresses GCaMP6 and RFP in ALM neuron. Left panel shows green fluorescence from GCaMP6m (left) and red fluorescence from RFP (right) in false colors. White boxes indicate location of AVM neuron and shows how algorithm tracks the neuron. Right graph shows the quantitative calcium trance and red circle indicates the current time point of video. Stimulus occurs at 10s (red dash line). The worm in this experiment was cultured 18-19h at 20 °C after hatch. 5x playback.

Movie 2: Calcium dynamics of PVD neuron to 1s posterior stimulation at 45 psi. Stimulus was delivered 10s after recording baseline of neuronal activity. The transgenic animal shown here expresses GCaMP6 and RFP in PVD neuron. The worm in this experiment was cultured 21-22h 20 °C after hatch. 5x playback.

Movie 3: Calcium dynamics of AVM neuron to 1s anterior stimulation at 50 psi. Stimulus was delivered 10s after recording baseline of neuronal activity. The transgenic animal shown here expresses GCaMP6 and RFP in AVM neuron. The worm in this experiment was cultured 22-23h 20 °C after hatch. 5x playback.
